# Ferrocene-Modified Nanoscale Covalent Organic Frameworks for Ferroptosis-Based Sonodynamic Therapy Inhibit Breast Cancer and Its Bone Metastasis

**DOI:** 10.34133/cbsystems.0490

**Published:** 2026-03-23

**Authors:** Ming Wu, Yiqing Zeng, JianGang Chen, Zhen Yang, Siyuan Song, Rongkai Yan, Taofik Al Hassan, Yan Zhang

**Affiliations:** ^1^ Department of Orthopaedics, Gongli Hospital of Pudong New Area, Shanghai 200135, China.; ^2^ Department of Ultrasound, the First Affiliated Hospital, Zhejiang University School of Medicine, Hangzhou, Zhejiang 310058, China.; ^3^ Postgraduate Training Base at Shanghai Gongli Hospital, Ningxia Medical University, Shanghai 750004, China.; ^4^ Baylor College of Medicine, Houston, TX 77030, USA.; ^5^ Department of Radiology, Ohio State University, Columbus, OH 43210, USA.

## Abstract

Breast cancer continues to be a marked risk to women’s health worldwide. In particular, bone metastasis stands as one of the leading causes of death among breast cancer patients. In recent years, therapies that reprogram the tumor’s immunosuppressive microenvironment have emerged as promising antimetastatic strategies. In this study, we developed a sonodynamic nanoplatform that simultaneously eradicates primary breast tumors via ferroptosis and apoptosis and blocks their spread to bone tissues. Ferrocene-grafted nanoconfined covalent organic frameworks (mCOFs) were synthesized by condensing aminoferrocene with micrometer-sized COFs, yielding a multifunctional agent capable of amplifying reactive oxygen species (ROS) production. Following ultrasound treatment, the mCOFs generated abundant ROS, while the ferrocene moieties catalyzed the Fenton-like conversion of endogenous H_2_O_2_ into cytotoxic •OH radicals. In vitro, ultrasound-activated mCOFs concurrently induced apoptosis and ferroptosis in breast cancer cells and triggered the robust release of immunogenic factors. In orthotopic mouse models, intravenously administered mCOFs preferentially accumulated in tumors; upon ultrasound exposure, these mCOFs markedly inhibited the growth of primary tumors, reprogrammed the immunosuppressive tumor microenvironment, and effectively reduced bone metastasis. This study proposes a versatile nanomedicine-based strategy that integrates sonodynamic therapy with immunomodulation to control breast cancer progression and bone metastasis, offering a broad approach for reducing metastatic disease.

## Introduction

Breast cancer is the most prevalent cancer among women globally [1,2]. Although extensive research over decades has resulted in a considerable increase in the 5-year survival rate of breast cancer patients, metastatic breast cancer, especially bone metastases, still presents a significant threat to both health and survival [3–5]. Conventional treatments for breast cancer primarily target the primary tumor and are often inadequate for effectively managing metastatic disease. Immunotherapy, by activating the systemic immune response to recognize and eliminate tumor cells, not only suppresses the growth of primary tumors but also demonstrates strong antitumor efficacy against metastases [6,7]. Clinical evidence further indicates that an immune-activated tumor microenvironment can inhibit tumor cell migration, thereby reducing the risk of metastasis [8–10]. Therefore, therapeutic strategies that eradicate breast cancer cells while also reversing tumor-induced immunosuppression could be valuable in improving patient outcomes.

Sonodynamic therapy (SDT) is an innovative treatment technique that utilizes ultrasound (US) to activate sonosensitizers, which subsequently lead to the generation of reactive oxygen species (ROS) inside cells through acoustic cavitation [11–14]. Compared to photodynamic therapy (PDT), SDT provides better tissue penetration and more precise control over both the spatial and temporal aspects, making it a promising option for the treatment of solid tumors [15–19]. Meanwhile, SDT has also been reported to reverse the immunosuppressive tumor microenvironment, thereby enhancing the efficacy of cancer immunotherapy [20,21]. Most multifunctional sonosensitizers developed so far have largely been based on inorganic materials (e.g., TiO_2_@Au, TiO_2_@SiO_2_, and TiO_1+*x*_), often containing complex architectures such as core–shell structures and heterojunctions to enhance therapeutic capabilities [22,23]. However, these inorganic systems frequently have drawbacks such as poor biodegradability and potential biotoxicity. Additionally, owing to their complex structures, their controlled, large-scale production is challenging [24–26]. In contrast, organic sonosensitizers, which are typically small molecules, offer superior biocompatibility and biosafety [27–29]. However, their structural versatility for multifunctionalization is often limited. Therefore, identifying biocompatible, multifunctional sonosensitizers that combine the advantages of both material classes is critical for expanding the clinical potential of SDT.

Covalent organic frameworks (COFs) have recently attracted attention due to their porous structures, tunable chemistry, and high biocompatibility [30–32]. As a result, these materials have emerged as promising candidates for applications such as drug delivery and in vivo imaging [33–35]. Notably, their ease of chemical modification also enables surface functionalization through the grafting of small molecules, polymers, and targeting ligands [36–38]. Hence, COFs can act as versatile multifunctional drug carriers with high biosafety. However, most of the building blocks used to prepare COFs are typically planar molecules. Hence, it is difficult to control their particle size during crystallization. Consequently, a top-down approach is commonly employed for the preparation. In this approach, bulk COFs (micrometer-sized) are chemically converted into nanoscale COFs (nano-COFs) [39–41]. Nevertheless, this method frequently introduces additional, often nonfunctional small molecules, which can hinder functionalization and introduce toxicity [42–44]. Thus, the identification of functional small molecules that can be employed in top-down synthesis to not only control COF particle size but also enhance therapeutic efficacy has become an important focus of research in the field of nano-COFs.

**Fig. 1.**
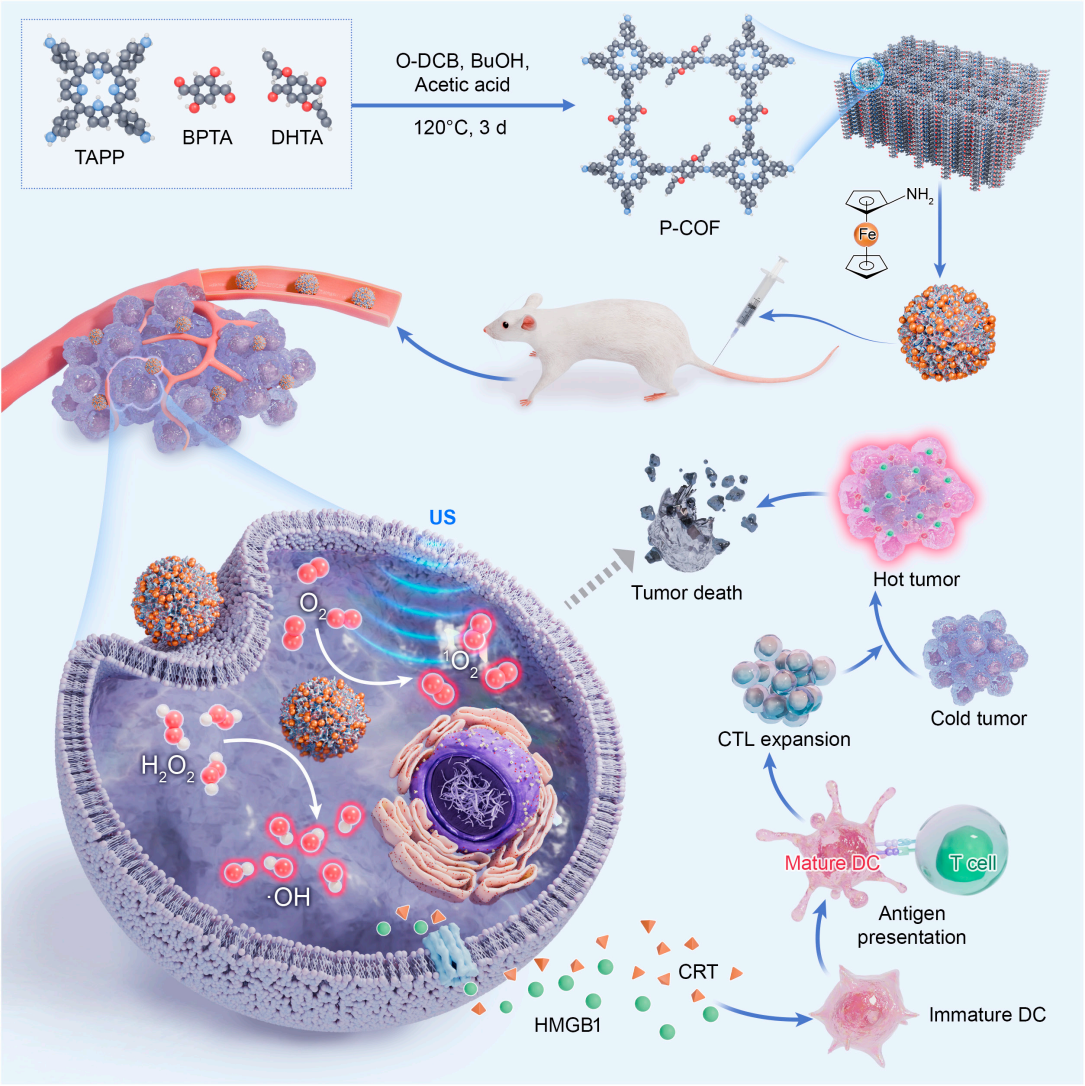
Schematic showing the preparation of mCOFs and their role in tumor growth inhibition. TAPP, tetra (p-amino-phenyl) porphyrin; BPTA, 2,5-bis(2-propynyloxy) terephthalaldehyde; DHTA, 2,5-dihydroxyterephthalaldehyde; O-DCB, o-dichlorobenzene; BuOH, n-butanol; HMGB1, high mobility group box 1 protein; CRT, calreticulin; DC, dendritic cell.

In this study, we developed a multifunctional nano-COF (mCOF) by reacting bulk COFs with aminoferrocene via a top-down strategy (Fig. 1). This approach preserves the crystallinity of the parent COF while yielding nanoscale particles. The incorporation of ferrocene moieties endows the mCOF with both sonodynamic and Fenton-like catalytic activities. Under US irradiation, mCOF facilitates the generation of singlet oxygen, while the Fe^2+^ centers in ferrocene catalyze the conversion of endogenous H_2_O_2_ in the tumor microenvironment into hydroxyl radicals via a Fenton-like reaction. This dual mechanism induces ferroptosis in tumor cells and enhances overall ROS-mediated cytotoxicity [45]. This ferroptosis-based SDT synergistically inhibits tumor growth. The ROS burst not only promotes apoptosis but also triggers immunogenic cell death (ICD), thereby activating a systemic antitumor immune response. Therefore, this study presents a safe and effective nanoplatform for synergistic ferroptosis-based SDT while also offering a rational strategy for the design of multifunctional sonosensitizers.

## Methods

### Synthesis of mCOFs

Synthesis of COF powder: tetra (p-amino-phenyl) porphyrin (TAPP) (0.022 mmol, 14.9 mg), 2,5-dihydroxyterephthalaldehyde (DHTA) (0.022 mmol, 3.7 mg), 2,5-bis(2-propynyloxy) terephthalaldehyde (BPTA) (0.022 mmol, 5.3775 mg), o-dichlorobenzene (0.5 ml), aqueous acetic acid (3 M, 0.1 ml), and n-butanol (0.5 ml) were mixed in a Pyrex tube. The tube underwent 3 freeze–pump–thaw cycles and was heated at 120 °C for 72 h. The product was isolated using a centrifuge and washed thrice with ethanol. For further purification, the precipitate was stirred with tetrahydrofuran (THF) for 24 h before filtration.

Synthesis of mCOF NPs: 10 mg of COF powder was dispersed in 10 ml of N,N-dimethylformamide (DMF). Then, a 0.2 M equivalent of aminoferrocene was added. The mixture was ultrasonicated for 30 min to obtain mCOF NPs. The mixture was centrifuged, and the precipitate was isolated and washed thrice with DMF.

### Cell culture

4T1 cells were cultured in complete high-glucose Dulbecco’s modified Eagle’s medium (DMEM), supplemented with 10% fetal bovine serum (FBS) and 1% penicillin–streptomycin. The cells were incubated at 37 °C with 5% CO_2_.

### Establishment of the bone metastasis model

To create the bone metastasis model, 4T1 tumor-bearing female BALB/c mice were first anesthetized by intraperitoneal injection of 0.1% pentobarbital sodium, followed by injection of 10^5^ 4T1 cells into the left tibia of each mouse.

### Statistical analysis

The data are presented as mean ± SD. Analysis of variance (ANOVA) was used to compare multiple groups, and *P* values were computed using Student’s *t* test. The significance levels were as follows: ***P < 0.001, **P < 0.01, *P < 0.05.

## Results and Discussion

### Synthesis and characterization of mCOFs

**Fig. 2.**
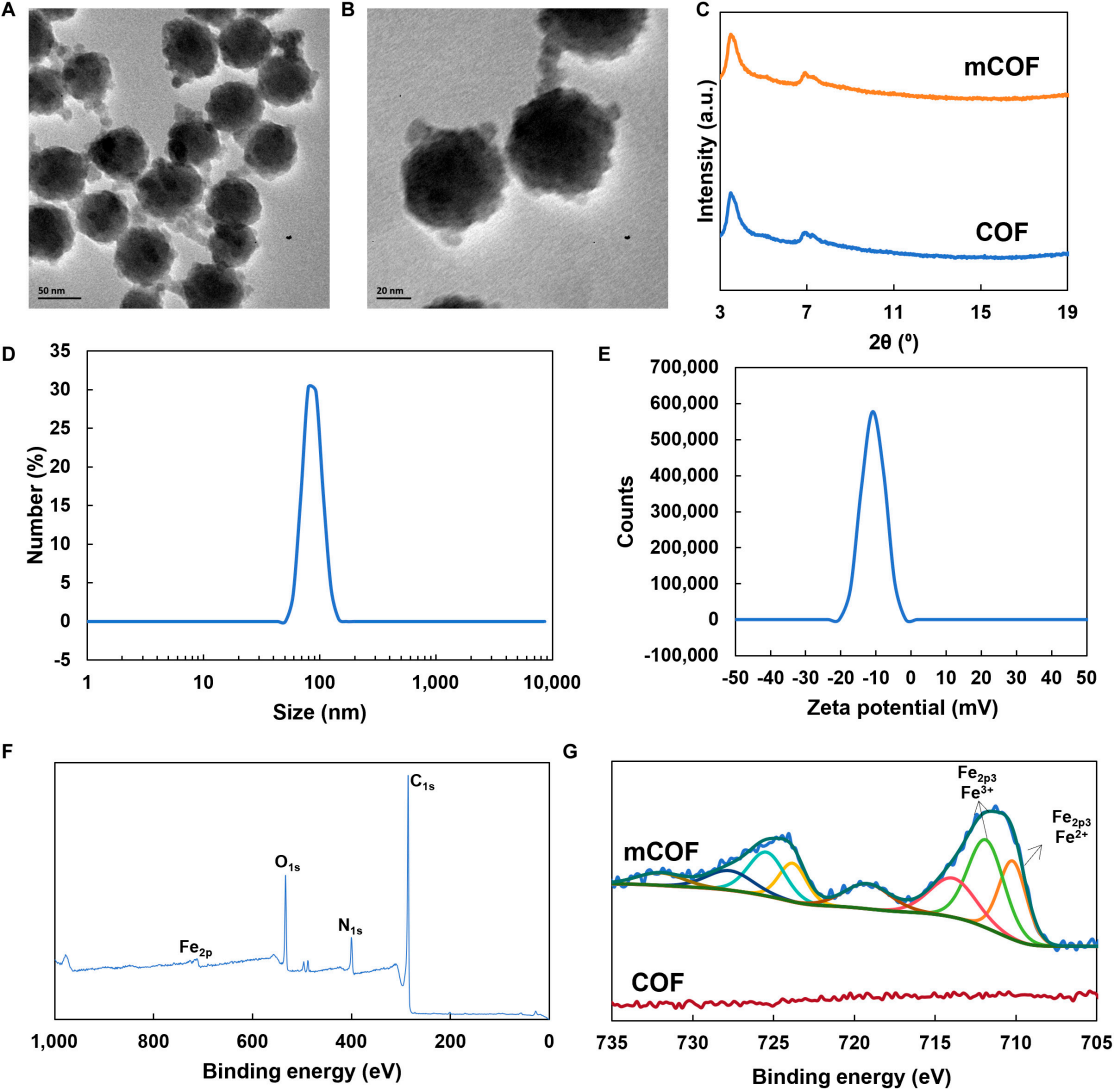
Characterization of mCOFs. (A and B) TEM images of mCOFs. (C) XRD pattern of mCOFs. (D) Particle size distribution and (E) zeta potential of mCOFs in an aqueous solution. (F) Full x-ray photoelectron spectrum of mCOFs. (G) Fe2p spectrum of both mCOFs and COFs.

First, we employed a solvothermal method to obtain powdered micrometer-scale COFs through heating at 120 °C (Fig. S1). Subsequently, the COF powder and aminoferrocene were mixed in DMF at a 5:1 molar ratio, ultrasonicated for 30 min, and then subjected to centrifugation to yield modified nano-COFs (mCOFs). Transmission electron microscopy (TEM) indicated that the resulting mCOFs had a relatively uniform particle size of approximately 50 nm (Fig. 2A and B). Subsequently, we conducted x-ray diffraction (XRD) measurements on COFs and mCOFs (Fig. 2C). The results showed that the crystal structure of COFs remained unchanged upon aminoferrocene functionalization. Thus, although COFs were transformed from a powdered form into a nanoparticle form, their overall crystalline structure remained unaffected. Moreover, the observed crystal structure was consistent with that reported in previous studies [46].

Fig. 2D shows the dispersibility and hydrodynamic size of mCOFs in phosphate-buffered saline (PBS). Notably, the mCOFs exhibited good dispersibility in PBS, showing an average hydrodynamic diameter of 87.3 nm ± 8.4 nm. Meanwhile, the zeta potential of mCOFs in PBS was also negative, averaging at −8.92 mV ± 1.34 mV (Fig. 2E). Notably, a negative zeta potential is favorable for the circulation of mCOFs in vivo. Further x-ray photoelectron spectroscopy showed that, unlike the COF powder, the synthesized mCOFs exhibited an absorption peak corresponding to Fe at 711.85 eV (Fig. 2F and Fig. S2). The additional analysis of absorption peaks (Fig. 2G) revealed that COF only displays noise signals in the 705- to 735-eV range, indicating the absence of Fe in COF. In contrast, mCOFs presented peaks corresponding to Fe2p3 Fe^2+^ and Fe2p3 Fe^3+^ at 710.2 and 711.85 eV, respectively, suggesting that aminoferrocene was incorporated into the COF framework during mCOF synthesis. In mCOF, the N species were mainly found in the form of C=N and C–N, and the corresponding peaks were located at 397.8 and 399.53 eV, respectively (Fig. S3). This finding suggests that mCOF is predominantly made up of nitrogen-based structures resembling porphyrins. The C species were mainly found in the form of C–C, C=O, and C–O bonds (284.8, 288.2, and 286.37 eV, respectively). These results confirmed that mCOFs were successfully synthesized and could be utilized for subsequent experiments.

**Fig. 3.**
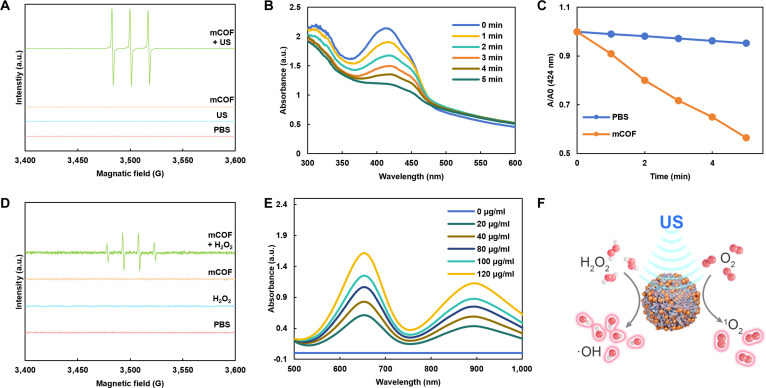
ROS generation capability of mCOFs. (A) EPR spectra of 1O2 in mCOF suspensions under varying conditions. (B) Time-dependent degradation of DPBF by mCOFs after ultrasonic treatment. (C) Normalized data demonstrating mCOFs’ ability to degrade DPBF with prolonged ultrasonication. (D) EPR spectra of •OH in mCOF suspensions under different conditions. (E) Absorption spectra of TMB relative to mCOF concentration. (F) Schematic.

To assess the sonodynamic properties of mCOFs, electron paramagnetic resonance (EPR) spectroscopy was conducted. Additionally, the production of singlet oxygen was examined under varying conditions using 2,2,6,6-tetramethylpiperidine (TEMP) as a spin-trapping probe. As shown in Fig. 3A, mCOFs produced the characteristic triplet signal of singlet oxygen (1:1:1) following US irradiation. To further quantify singlet oxygen generation, we examined the US-triggered degradation of 1,3-diphenylisobenzofuran (DPBF) by mCOFs (Fig. 3B). Compared with the PBS group, where only 4.7% of DPBF was degraded following US treatment (Fig. S4), the mCOF group achieved 43.5% DPBF degradation under the same conditions (Fig. 3C). These results demonstrated that mCOFs can effectively generate singlet oxygen following ultrasonication. Subsequently, EPR tests using 5,5-dimethyl-1-pyrroline N-oxide (DMPO) as the spin-trapping agent detected the distinct quartet signal (1:2:2:1) associated with hydroxyl radicals when both mCOFs and H_2_O_2_ were present (Fig. 3D). Next, 3,3′,5,5′-tetramethylbenzidine (TMB) served as an indicator to measure hydroxyl radical generation. Thereafter, TMB was used as an indicator to evaluate hydroxyl radical production. As shown in Fig. 3E, the colorless TMB was oxidized to blue ox-TMB, and its characteristic absorption signal at 652 nm became increasingly stronger as the mCOF concentration increased. This indicated that in an H_2_O_2_-rich environment, mCOFs can effectively generate hydroxyl radicals, thereby inducing tumor damage. Collectively, the findings suggested that mCOFs could produce singlet oxygen following ultrasonication, with the iron centers in ferrocene decomposing H_2_O_2_ to generate hydroxyl radicals in the H_2_O_2_-rich tumor microenvironment (Fig. 3F). The combination of these 2 types of ROS was expected to yield a potent antitumor effect [47,48].

### In vitro tumor cell death due to mCOF treatment and mechanistic analysis

**Fig. 4.**
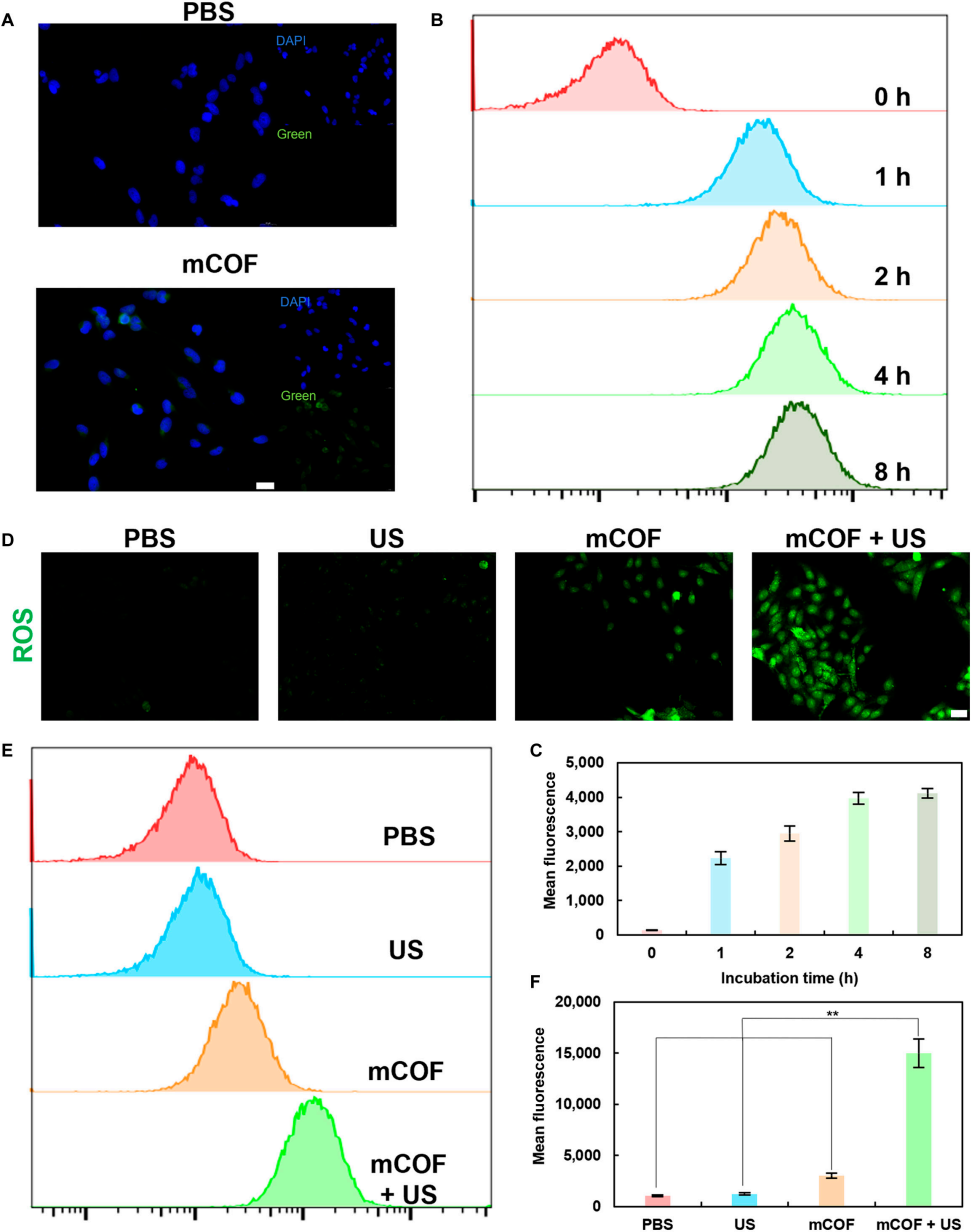
Entry of mCOFs into tumor cells and ROS generation in response to ultrasonic treatment. (A) Confocal images showing mCOFs in 4T1 cells after 4 h of coincubation (scale bar, 20 μm). (B) Flow cytometry analysis and (C) quantification of mCOFs in 4T1 cells after varying incubation periods. (D) Fluorescence images of ROS production in cells under different conditions (scale bar, 100 μm). (E) Flow cytometry and (F) quantification of ROS production in mCOF-treated cells (*n* = 3, mean ± SD).

To validate our experimental findings regarding the robust antitumor potential of mCOFs, we first examined their cellular uptake in vitro based on the inherent fluorescence of the porphyrin structures within mCOFs. After 4 h of co-incubation, pronounced porphyrin-derived fluorescence was observed within 4T1 cells (Fig. 4A). The fluorescence was distributed uniformly throughout, indicating that mCOFs efficiently enter 4T1 cells and disperse within the cytoplasm. This localization was conducive to the breakdown of H_2_O_2_ into hydroxyl radicals and the production of singlet oxygen following ultrasonication, which could promote tumor cell death. Subsequently, flow cytometric analysis (Fig. 4B and C) revealed that the amount of mCOFs internalized by cells increased progressively with the co-incubation period, peaking at 4 h. This finding also suggested that 4 h was the optimal time point for ultrasonication.

This result also indicated that 4 h was the optimal duration for ultrasonication. Following this, the potential of mCOFs to generate intracellular ROS was assessed (Fig. 4C). After 4 h of co-incubation, mCOFs reacted with H_2_O_2_ to produce hydroxyl radicals, and under additional US treatment, large amounts of singlet oxygen were generated. Flow cytometry showed that intracellular ROS levels increased by 2.89-fold and 12.22-fold after treatment with mCOF and mCOF + US, respectively (Fig. 4E and F). The anticipated increase in ROS production was expected to reduce the viability of tumor cells.

**Fig. 5.**
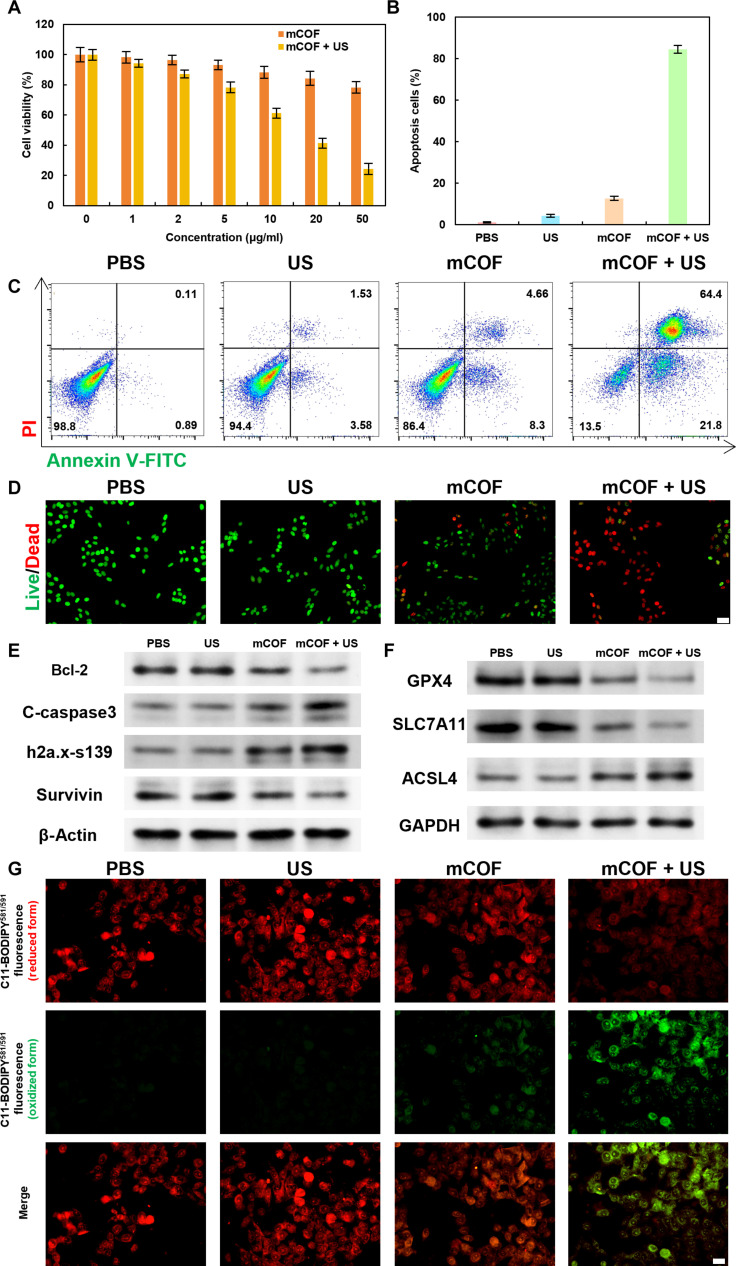
Ferroptosis-based tumoricidal effect of mCOFs on 4T1 cells after SDT. (A) mCOF-induced cell killing with or without US treatment at varying concentrations. (B) Quantification and (C) flow cytometric analysis of apoptosis in 4T1 cells after treatments. (D) Representative calcein-AM/PI staining images in 4T1 cells (scale bar, 100 μm). Western blot showing expression of (E) apoptosis-related proteins and (F) ferroptosis-related proteins. (G) CLSM images of C11-BODIPY581/591 fluorescence distribution in 4T1 cells (scale bar, 50 μm).

Given the excellent sonodynamic and chemodynamic performance of mCOFs, their in vitro therapeutic efficacy against 4T1 tumor cells was examined at the cellular level. First, cell inhibition was measured using the Cell Counting Kit-8 (CCK-8) assay. As shown in Fig. 5A, the viability of 4T1 tumor cells decreased as the concentration of mCOF increased, reaching 78.3% at a concentration of 50 μg/ml, confirming that mCOFs can specifically kill tumor cells by catalyzing the conversion of H_2_O_2_ to •OH. After US irradiation (1 W/cm^2^, 1 MHz, 5 min), cell viability exhibited a more pronounced decrease with increasing mCOF concentrations. Following treatment with 50 μg/ml mCOF combined with US irradiation (5 min), cell viability reduced to only 24.3%. This marked a 54% decrease when compared to mCOF treatment alone, without US irradiation, highlighting the remarkable sonocatalytic antitumor activity of the fabricated platform. Flow cytometry apoptosis assays showed that the apoptosis rates in the mCOF and mCOF + US groups were 12.66% and 84.51%, respectively, clearly demonstrating that combination therapy effectively induces tumor cell apoptosis (Fig. 5B and C). Subsequently, calcein-AM/propidium iodide (PI) double staining further demonstrated the highest red fluorescence in the mCOF + US group, followed by the mCOF group (Fig. 5D and Fig. S6). These findings also indicated that the ROS generated due to synergistic sonodynamic and chemodynamic catalysis is highly cytotoxic to tumor cells, with sonocatalysis having a more prominent contribution.

The increased ROS production was expected to reduce the viability of tumor cells. Initially, cell inhibition was tested using the CCK-8 assay. Further Western blot analysis (Fig. 5E and Table S1) showed that, after mCOF treatment combined with US irradiation, anti-apoptotic proteins such as Bcl-2 (from 0.727 to 0.379) and Survivin (from 0.536 to 0.434) were notably down-regulated in 4T1 cells compared to the PBS group. On the other hand, pro-apoptotic proteins like C-caspase-3 (from 0.286 to 0.561) and H2a.x-s139 (from 0.727 to 0.379) were significantly up-regulated. These results suggest that the combined treatment of mCOF and US induces tumoricidal effects by regulating apoptosis. Protein expression levels of GPX4, SLC7A11, and ACSL4 in 4T1 cells (Fig. 5F) were also analyzed. After mCOF and US treatment, GPX4 (from 0.976,7 to 0.333,5) and SLC7A11 (from 0.799 to 0.237) levels were significantly reduced, whereas ACSL4 (from 0.851 to 0.150,6) expression was greatly increased. This further confirmed the induction of ferroptosis in 4T1 cells [49]. Confocal laser scanning microscopy (CLSM) images shown in Fig. 5G illustrated the distribution of C11-BODIPY581/591 (oxidized form) fluorescence in 4T1 cells after the treatments. Notably, the 4T1 cells displayed a low level of lipid peroxidation after treatment with PBS and US, a moderate level following treatment with mCOF, and a high level after mCOF + US treatment. These findings demonstrated that mCOF, when combined with US irradiation, enhances lipid peroxidation in 4T1 cells, promoting ferroptosis, thereby further enhancing the predominant apoptosis and further inhibiting tumor cell growth.

### Maturation of DCs following mCOF treatment in vitro

**Fig. 6.**
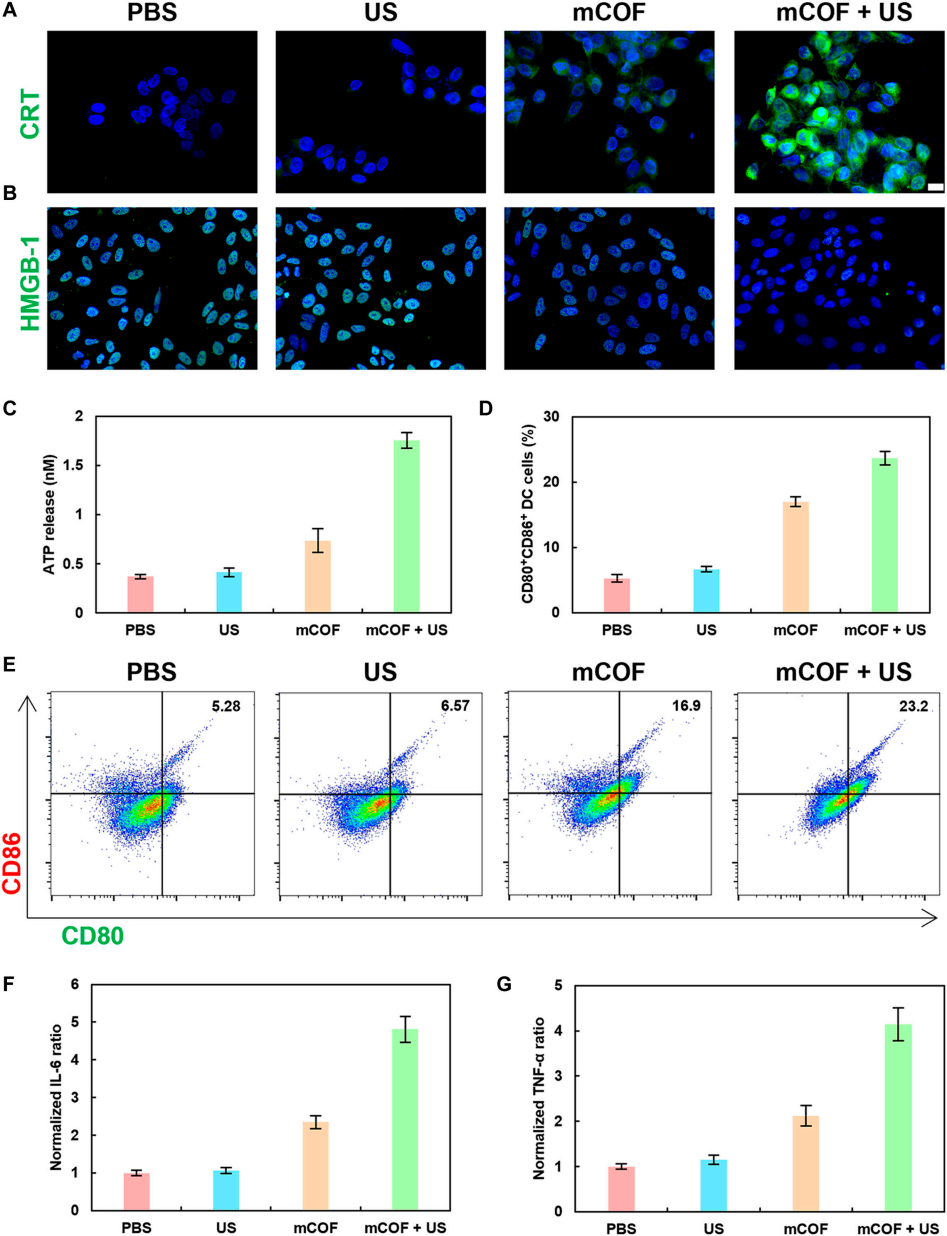
Immune factor release and DC activation in vitro after mCOF treatment of 4T1 cells. Immunofluorescence staining for (A) CRT and (B) HMGB1 (scale bar, 50 μm) in 4T1 cells after various treatments. (C) ATP release from 4T1 cells after treatments. (D) Quantification and (E) flow cytometry analysis of DC activation. (F) IL-6 and (G) TNF-α levels in DCs after treatments (*n* = 3, mean ± SD).

Our prior experiments demonstrated that US-activated mCOFs induce apoptosis and produce tumoricidal effects. As apoptotic tumor cells can promote dendritic cell (DC) maturation and amplify antitumor immunity through cytokine release, we quantified the levels of calreticulin (CRT), high mobility group box 1 protein (HMGB1), and adenosine triphosphate (ATP) in 4T1 cells following various treatments (Fig. 6A to C and Figs. S7 and S8). Confocal microscopy revealed the significant down-regulation of HMGB1 and up-regulation of CRT after mCOF + US treatment, and the content of released ATP increased from 0.37 nM to 1.75 nM. These findings indicated that HMGB1, CRT, and ATP were released into the culture medium following treatment. Thereafter, the ability of this conditioned medium to promote DC maturation in vitro was tested. Notably, flow cytometry showed that the conditioned medium enhanced DC maturation in vitro (increased from 5.27% to 23.6%) (Fig. 6D and E and Fig. S9) and increased interleukin-6 (IL-6) and tumor necrosis factor-α (TNF-α) levels by 3.81- and 3.14-fold, respectively. These findings suggested that the apoptosis induced by mCOF + US can promote DC maturation via HMGB1 and CRT release and thereby enhance immunotherapeutic efficacy.

### In vivo cancer inhibition

**Fig. 7.**
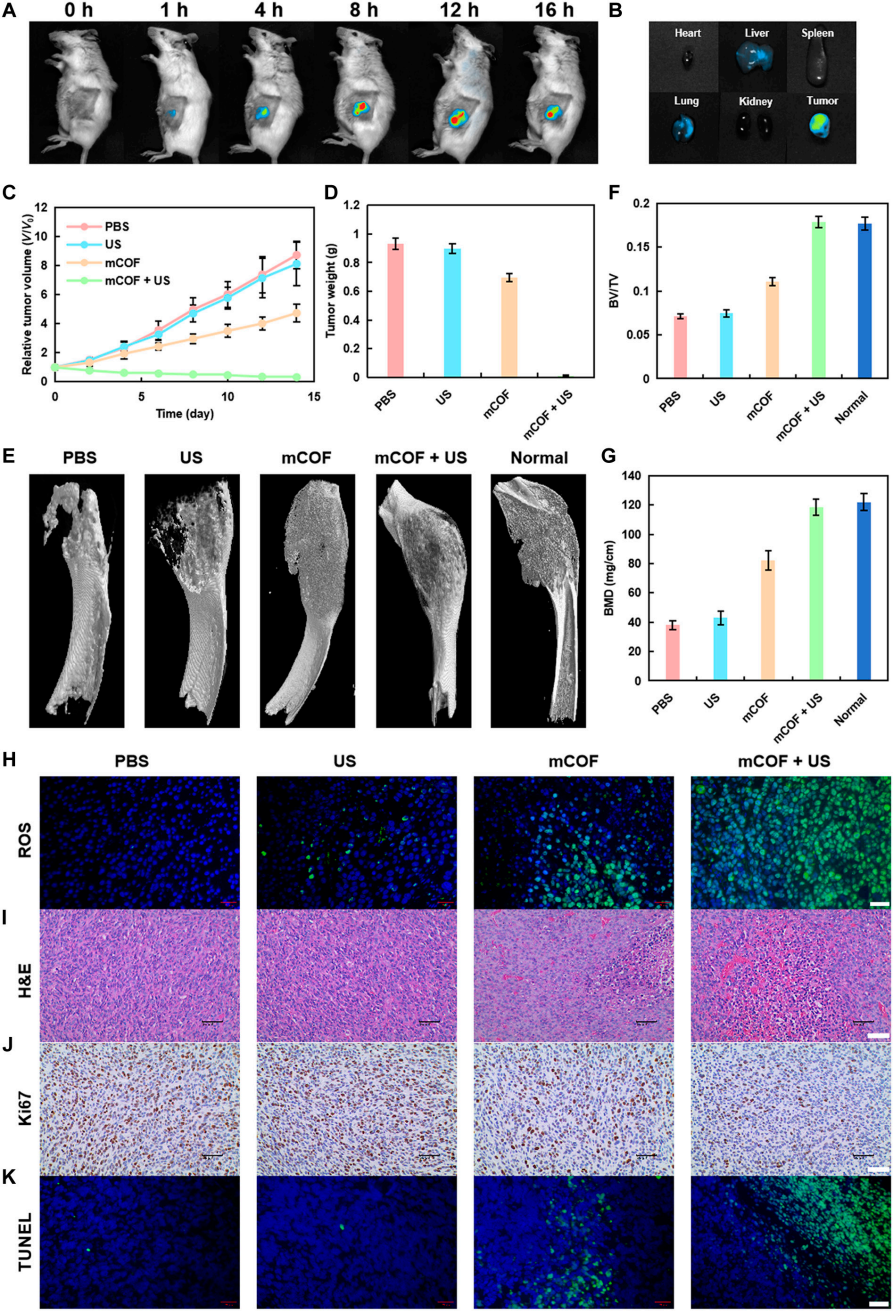
In vivo distribution of mCOFs and tumor inhibition evaluation. (A) Time-dependent in vivo distribution of mCOFs in 4T1 tumor-bearing mice after tail vein injection. (B) Fluorescence imaging of key organs 12 h after mCOF injection. (C) Tumor volume changes in mice receiving different treatments over 14 d (*n* = 5, mean ± SD). (D) Tumor weight of mice receiving treatments for 14 d (*n* = 5, mean ± SD). (E) Micro-CT 3D reconstruction of mouse tibias after treatment. (F) BV/TV and (G) BMD values (*n* = 5, mean ± SD). (H) ROS levels in tumors from treated mice (scale bar, 20 μm). (I) H&E staining (scale bar, 50 μm), (J) Ki67 staining (scale bar, 50 μm), and (K) TUNEL staining (scale bar, 20 μm) of breast tumors after treatments.

Finally, a 4T1 breast cancer bone metastasis model was created to confirm the effectiveness of mCOF combined with US in treating bone metastasis. To identify the best timing for US treatment, the biodistribution of mCOFs was measured after tail vein injection (Fig. 7A and B). Tumor accumulation was highest at 12 h post-injection, indicating that 12 h was the optimal time for US irradiation. The weight of tumor-bearing mice was comparable across groups during treatment (Fig. S10), suggesting that the treatment had minimal adverse effects. Meanwhile, no obvious damage in normal tissues could be observed after different treatments (Fig. S11). The relative tumor volumes (*V*/*V*_0_) were tracked across different groups during treatment. Notably, tumors in the control and US groups grew quite rapidly, whereas treatment with mCOF alone modestly delayed tumor growth. By contrast, the mCOF + US group exhibited marked tumor growth inhibition, suggesting that ferroptosis and sonodynamic activity led to amplified antitumor effects (Fig. 7C). Tumors collected from each treatment group at the end of the experimental period further verified the enhanced antitumor activity of mCOF combined with US (Fig. 7D).

Microcomputed tomography (micro-CT) was subsequently used to assess tibial morphology and structure across treatment groups (Fig. 7E). Three-dimensional reconstructions revealed that the mCOF + US group exhibited reduced osteolytic destruction when compared to the PBS group. To further evaluate the protective effects of treatment, bone microarchitectural parameters were quantified (Fig. 7F and G). The mCOF + US group showed significantly higher relative bone volume (BV/TV) and bone mineral density (BMD) than the other treatment groups. Collectively, these data indicated that mCOF combined with US improves bone architecture by inhibiting tumor growth and osteoclast differentiation due to its dual-targeting effects on primary and metastatic tumors.

Thereafter, tumor tissues from each group were sectioned and histologically analyzed. ROS fluorescence revealed abundant ROS generation following US in the mCOF + US group, consistent with effective tumor cell death and tumor growth inhibition (Fig. 7H and Fig. S12). Hematoxylin and eosin (H&E) staining showed a greater tumor cell number and density in the PBS and US groups, whereas the mCOF and mCOF + US groups displayed extensive regions of tumor necrosis. This necrosis was more pronounced in the mCOF + US group than in the mCOF group (Fig. 7I). Notably, Ki67 and TUNEL (terminal deoxynucleotidyl transferase–mediated deoxyuridine triphosphate nick end labeling) staining further confirmed the strong tumoricidal and growth inhibitory effects in the mCOF and mCOF + US groups, with the therapeutic effects being stronger in the latter (Fig. 7J and K).

**Fig. 8.**
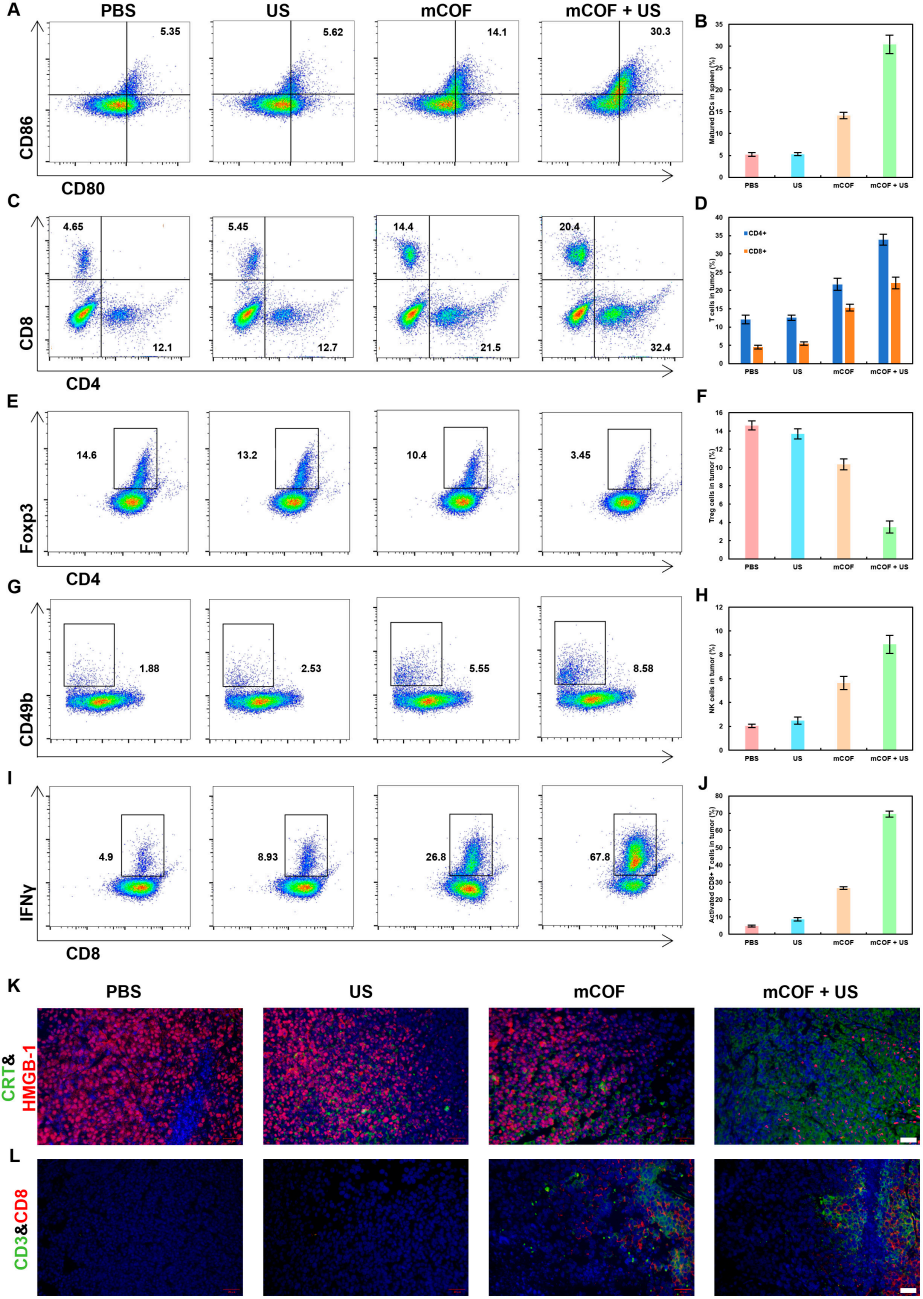
Immune responses induced by mCOF treatment in vivo. (A) Flow cytometric analysis and (B) quantification of mature DCs (CD45^+^ CD11b^+^ MHCII^+^ CD80^+^ CD86^+^) in the draining lymph nodes of tumor-bearing mice. (C) Flow cytometric analysis and (D) quantification of CD8^+^ T cells (CD45^+^ CD3^+^ CD8^+^) and CD4^+^ T cells (CD45^+^ CD3^+^ CD4^+^) in tumor tissues. (E) Flow cytometric analysis and (F) quantification of Treg cells (CD45^+^ CD3^+^ CD4^+^ FoxP3^+^) in tumors. (G) Flow cytometric analysis and (H) quantification of NK cells (CD45^+^ CD3^−^ CD49b^+^) in tumors. (I) Flow cytometric analysis and (J) quantification of activated CD8^+^ T cells (CD45^+^ CD3^+^ CD8^+^ IFN-γ^+^) in tumors. (*n* = 3, mean ± SD). Fluorescence images showing (K) CRT and HMGB1 expression and (L) CD3 and CD8 expression at tumor sites in 4T1 tumor-bearing mice after treatments (scale bar, 20 μm).

To investigate the immunomodulatory effects of the treatment, the abundance of various cell types in mouse lymph nodes and tumors was measured (Fig. 8A to J). Initially, the mature DCs in the lymphatic vessels were counted. The number of mature DCs was significantly higher in the mCOF and mCOF + US groups compared to the other 3 groups. Moreover, SDT increased DC numbers, thereby promoting immune responses. Subsequently, the CD4^+^ and CD8^+^ T cells in tumors were also analyzed. mCOFs effectively increased intratumoral CD4^+^ and CD8^+^ T cells, and SDT may also boost immunotherapy by disrupting the immunosuppressive tumor microenvironment. A notable increase in natural killer (NK) cells and interferon-γ (IFN-γ)-positive CD8^+^ T cells was observed after ferroptosis induction and sonodynamic treatment, further supporting the increased presence of active immune cells in the tumor tissues. This may result in stronger tumoricidal activity and enhanced immunotherapy outcomes. The expression of CRT and HMGB1 in tumor cells was also examined across groups (Fig. 8K and Fig. S13). Notably, CRT levels increased significantly after treatment, whereas HMGB1 levels decreased markedly following mCOF and US treatment, consistent with in vitro findings. These results demonstrated that mCOF + US can promote the release of immune factors, thereby enhancing the efficacy of immunotherapy. Immunofluorescence analysis of CD3 and CD8 expression revealed their up-regulation after SDT, potentially triggering immune effects that inhibit tumor growth (Fig. 8L and Fig. S14). Overall, the therapeutic benefits in mice treated with the mCOF + US regimen were attributable to the effects of SDT, which directly killed tumor cells, activated the immune system, and alleviated immunosuppression in the tumor microenvironment. Compared with previously reported inorganic sonosensitizers, mCOF exhibits higher biocompatibility and potential biodegradability while maintaining a high yield of ROS, thereby reducing potential safety concerns associated with inorganic sonosensitizers. In comparison with organic sonosensitizers, mCOF itself can act as a carrier to accumulate in tumor tissues without the need for additional excipients, thus avoiding extra biocompatibility considerations. This therapeutic regimen proved to be even more effective at eliminating tumor cells and inhibiting tumor growth when compared to other treatment protocols.

## Conclusion

In this study, aminoferrocene was covalently integrated with a crystalline COF to yield a multifunctional, nanoscale sonosensitizer (mCOF) while preserving the original crystalline lattice. Upon US irradiation, mCOF underwent improved charge-carrier separation and transfer, which boosted ROS production from the excited framework. Concurrently, the ferrous centers catalyzed a Fe^2+^-mediated Fenton-like reaction in the H_2_O_2_-rich tumor microenvironment to generate •OH and promote lipid peroxidation. These processes collectively diminished the antioxidant buffering capacity of the tumor microenvironment (TME) and sensitized tumors to oxidative injury. Importantly, mCOF + US elicited ICD, characterized by CRT increased exposure in cell and more HMGB1 and ATP were released to the microenvironment, which primed DCs and activated systemic antitumor immunity. Consistent with these mechanisms, mCOF significantly suppressed tumor growth in vitro and in vivo following US irradiation. Overall, the multifunctional nanoscale design of mCOFs developed in this study offers a robust route for optimizing sonosensitizer performance and coupling sonodynamic and ferroptotic effects. The results of this research could assist in the development of nanomaterials for multimodal cancer treatments that offer lasting and systemic therapeutic benefits.

## Ethical Approval

All animal experiments were carried out after the approval of experimental protocols by the Institutional Animal Care and Use Committee (IACUC) of The First Affiliated Hospital, Zhejiang University School of Medicine (Reference Number: 2025 SHI DONG No. 030).

## Data Availability

All data are provided within the manuscript or supplementary information files.
