# Supplementary material for: Ferrocene-Modified Nanoscale Covalent Organic Frameworks for Ferroptosis-Based Sonodynamic Therapy Inhibit Breast Cancer and Its Bone Metastasis
Source: Cyborg Bionic Syst. 2026 Mar 23;7:0490. doi: 10.34133/cbsystems.0490 (PMC13006732; doi:10.34133/cbsystems.0490)
Supplement: Supplementary 1 — Materials and Methods Figs. S1 to S14 Tables S1 and S2 [file cbsystems.0490.f1.docx]

# Ferrocene-Modified Nanoscale Covalent Organic Frameworks for Ferroptosis-Based Sonodynamic Therapy Inhibit Breast Cancer and Its Bone Metastasis

Ming Wu^1,#^, Yiqing Zeng^2,#^, JianGang Chen^3^, Zhen Yang^1^, Siyuan Song^4^, Rongkai Yan^5^, Taofik Al Hassan^5^, Yan Zhang^1,*^

1. Department of Orthopaedics, Gongli Hospital of Pudong New Area, Shanghai, China
2. Department of Ultrasound, the First Affiliated Hospital, Zhejiang University School of Medicine, Hangzhou, Zhejiang, China
3. Postgraduate Training Base at Shanghai Gongli Hospital, Ningxia Medical University, Shanghai, China
4. Baylor College of Medicine, Houston, TX, USA
5. Ohio State University, Department of Radiology, Columbus, Ohio, USA

#, Those authors contributed equal to this work.

*, Corresponding author: Yan Zhang (rmyyzh@126.com).

**Experimental Section**

**1. Materials**

The materials were all purchased from Tansoole, the reagent kits were obtained from Solarbio, and the antibodies were purchased from Biolegend. Nude mice (5 weeks, around 16g) were purchased from Zhejiang University.

**2. Characterization**

Transmission electron microscopy (TEM) images, energy dispersive X-ray spectroscopy (EDS) and corresponding elemental mapping, High-resolution TEM (HRTEM) images, and selected area electron diffraction (SAED) patterns were applied to observe the particle size, morphology, element distribution, and crystal structure of HMC by using transmission electron microscopy (Talos F200x, USA). The crystal information of materials was assessed by X-ray diffractometer (XRD, D8 ADVANCE, BRUKER AXS GMBH, Karlsruhe, Germany), Dynamic Light Scattering (DLS, Zetasizer Advance ZSU3305, Malvern Instruments Limited, UK) was applied to measure the average hydrated particle size and zeta potential of materials. The absorption spectrum of TBNs was obtained through UV–vis–NIR spectrophotometric (Cary5000, Agilent, California, UK). The chemical composition and element status of materials were analyzed by X-ray photoelectron spectroscopy (XPS, AXIS Ultra DLD, Kratos, UK).

**3. In Vitro Detection of Reactive Oxygen Species (ROS)**

The ROS probe DPBF was used to detect ROS generated by BCOP under ultrasound irradiation. A mixture of mCOF and DPBF (80 µM, 2 mL) was subjected to ultrasound irradiation (1 MHz, 1 W cm^−2^) for varying durations. Absorbance changes were detected using an absorption spectrophotometer. The characteristic absorption peak of DPBF is at 421 nm. Absorbance at 421 nm for different time points was normalized using the formula: A = A_t min_ / A_0 min_ × 100%, where A_t min_ and A_0 min_ are the absorbances at 421 nm before and after t minutes of ultrasound irradiation, respectively.

**4. Detection of Hydroxyl Radical Generation for Chemodynamic Therapy**

TMB (3,3’,5,5’-tetramethylbenzidine) was employed as an indicator for •OH detection. mCOF (0, 20, 40, 80, 100, and 120 µg mL^−1^), H_2_O_2_ (10 mM), and TMB (0.8 mM) were mixed in 3 mL of PBS. After incubation at room temperature for 8 min, the absorbance of ox-TMB at 655 nm and 895 nm was measured to evaluate the chemodynamic performance.

**5. Electron Spin Resonance Detection of ROS**

DMPO and TEMP were used as spin traps to detect ROS generation. BCOP (100 µg mL^−1^) was mixed with DMPO or TEMP. After ultrasound irradiation (1 MHz, 1 W cm^−2^, 5 min) or added H_2_O_2_, ESR signals were detected using an electron paramagnetic resonance spectrometer.

**6. Cell Viability Assessment**

Cell viability was assessed using the Cell Counting Kit-8 (CCK-8) assay. 4T1 cells were seeded in 96-well plates at a density of 1×10^3^ cells/well. After 12 h, cells were treated under different conditions and incubated for 4 h. Cells were then washed with fresh RPMI-1640 medium and subjected to ultrasound irradiation (1.0 MHz, 17 W cm^−2^, 5 min). After further incubation for 20 h, cells were washed with PBS, and CCK-8 reagent was added for 40 min incubation. Absorbance at 450 nm was measured using a microplate reader. Cell viability was calculated as: Cell viability (%) = (Absorbance of treated group) / (Average absorbance of control group) × 100%.

**7. Detection of Intracellular ROS Levels**

The ROS probe 2’,7’-dichlorodihydrofluorescein diacetate (DCFH-DA, Beyotime, China) was used to detect intracellular ROS levels. 4T1 cells were seeded in 12-well plates at a density of 1×10^4^ cells/well and cultured for 24 h. After incubation with mCOF (100 µg mL^−1^), cells were treated with ultrasound irradiation (1.0 MHz, 1 W cm^−2^, 5 min). Cells were then incubated with DCFH-DA in the dark for 30 min according to the manufacturer’s instructions and washed with PBS. Intracellular green fluorescence was observed using a fluorescence microscope.

**8. Live/Dead Cell Staining**

Live and dead cells were stained using Calcein-AM and propidium iodide (PI) (Sigma-Aldrich, USA), respectively. 4T1 cells were seeded in 24-well plates and cultured for 12 h. Cells were then treated with PBS/RPMI-1640 solution containing mCOF for 4 h, and relevant groups were exposed to ultrasound irradiation (1.0 MHz, 1 W cm^−2^, 5 min). After further incubation for 20 h, cells were incubated with Calcein-AM/PI solution for 20 min. After washing three times with PBS, green fluorescence (Calcein-AM) and red fluorescence (PI) were captured and imaged using a fluorescence microscope.

**9. Western Immunoblotting**

4T1 cell-derived proteins were extracted using lysis buffer supplemented with protease inhibitors. An equivalent amount of protein was resolved on a 12% SDS-PAGE gel and subsequently transferred onto a PVDF membrane. After blocking the membrane in 5% non-fat milk dissolved in TBST for 1 hour, it was incubated overnight at 4 ℃ with primary antibodies targeting specific proteins, including Bcl-2, C-caspase3, h2x.x-s139, Survivin, β-actin, GPX4, SLC7A11, ACSL4 and GAPDH. Following three washes with Tris-Buffered Saline with Tween 20 (TBST), the membrane underwent a 1-hour incubation with corresponding secondary antibodies at room temperature, followed by additional TBST washes. Ultimately, the protein bands were detected using an enhanced chemiluminescence detection system.

**10. Evaluation of intracellular lipid peroxides (LPO) level:**

For CLSM analysis, 4T1 cells (1.0 × 105 cells) were first seeded into confocal dishes, and incubated overnight to achieve adherence. Next, the culture media were replaced with fresh one without or with Ce6@MHFe and different treatment. After 24 h of incubation, the cells were washed with PBS, fixed with 4.0 % of paraformaldehyde for 30 min, permeabilized with 0.10 % Triton X-100 for 5.0 min, blocked with 1.0 % BSA for 30 min. The cells were then stained with DAPI for 30 min, and BODIPY-C11^581/591^ (5.0 μM) for 30 min. Finally, the cells were observed by CLSM.

**11. CRT and HMGB1 Detection**

Calreticulin (CRT) and high-mobility group box 1 (HMGB1) levels were assessed by immunofluorescence staining. Cells were seeded in 12-well plates at a density of 1×10^4^ cells/well and cultured for 24 h. Groups were incubated with mCOF (100 µg mL^−1^) for 24 h, followed by group-specific ultrasound irradiation (1.0 MHz, 17 W cm^−2^, 5 min). CRT staining was performed at 6 h post-treatment; HMGB1 staining was performed at 12 h post-treatment. Cells were washed with PBS, fixed with 4% paraformaldehyde for 20 min, and permeabilized with 0.1% Triton X-100 (Servicebio, China) for 10 min. After blocking with 10% bovine serum albumin (BSA, Servicebio, China), cells were incubated with primary antibodies against CRT and HMGB1 (Servicebio, China) at room temperature for 2 h, followed by incubation with Alexa Fluor 488-conjugated secondary antibody for 1 h. Finally, nuclei were counterstained with DAPI for 10 min, and samples were observed under a fluorescence microscope.

**12. Tumor Model**

A 4T1 tumor model was established in 5-week-old female Balb/c mice. Mice were purchased from the Laboratory Animal Management Department of the Shanghai Institute of Planned Parenthood Research. A 4T1 cell suspension (100 μL, 2×10^6^ cells mL^−1^) was injected subcutaneously into the right posterior armpit as the tumor.

**13. Assessment of different groups of immune cells**

To assess the immune response induced by different therapy, various tissues, including the spleen, tumor-draining lymph nodes, primary tumor, distal tumor, and recurrent tumor, were surgically excised from mice across different experimental groups. Following digestion with collagenase IV (0.3 mg/mL) at 37°C for 1 h, single-cell suspensions were obtained through filtration using a 70 μm mesh. Subsequently, the harvested cells underwent CD16/CD32 antibody blocking for 15 min, followed by staining with eBioscience™ Fixable Viability Dye eFluor™ 506 for 15 min at 4℃.

The collected cells were then incubated with anti-CD45, anti-CD3, and anti-CD8 antibodies for evaluating the CD8+ T cell content within the tumors, employing standard flow cytometry protocols. Additionally, the cells were stained with anti-CD45, anti-CD3, anti-CD4, and anti-FoxP3 antibodies to assess the proportion of Treg T cells. For evaluating the proportion of NK cells, the collected cells were incubated with anti-CD45, anti-CD3, and anti-NK1.1+ antibodies. The frequency of mature DCs in lymph nodes was examined by flow cytometry after staining with anti-CD45, anti-MHCII, anti-CD11b, anti-CD80, and anti-CD86.

**14. Histological analysis**

Major organs, including the liver, spleen, kidney, heart, lungs, and brain along with tumor tissues from mice in each treatment group, were systematically harvested and preserved in 4% paraformaldehyde. Prior to electron microscopy, tissue samples underwent hematoxylin and eosin (H&E), terminal deoxynucleotidyl transferase dUTP nick end labeling (TUNEL), and Ki67 staining for detailed histological examination.


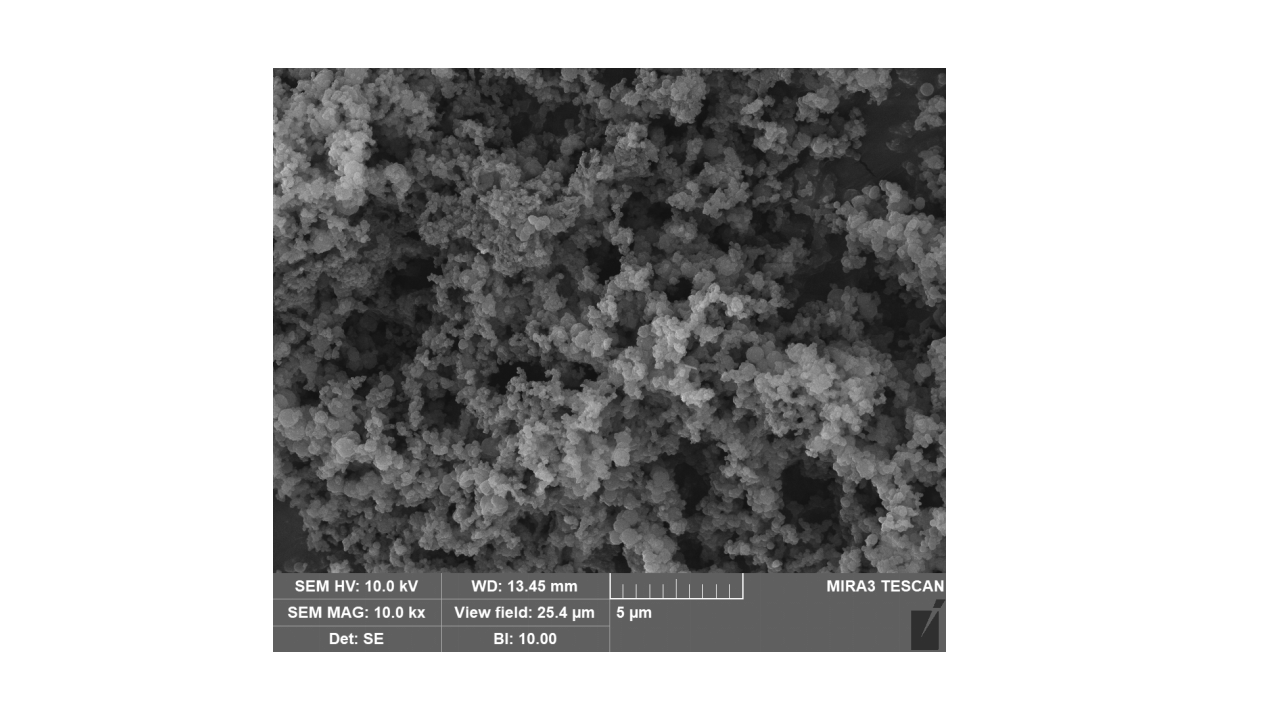


Figure S1 SEM image of synthesized micro-size COF.


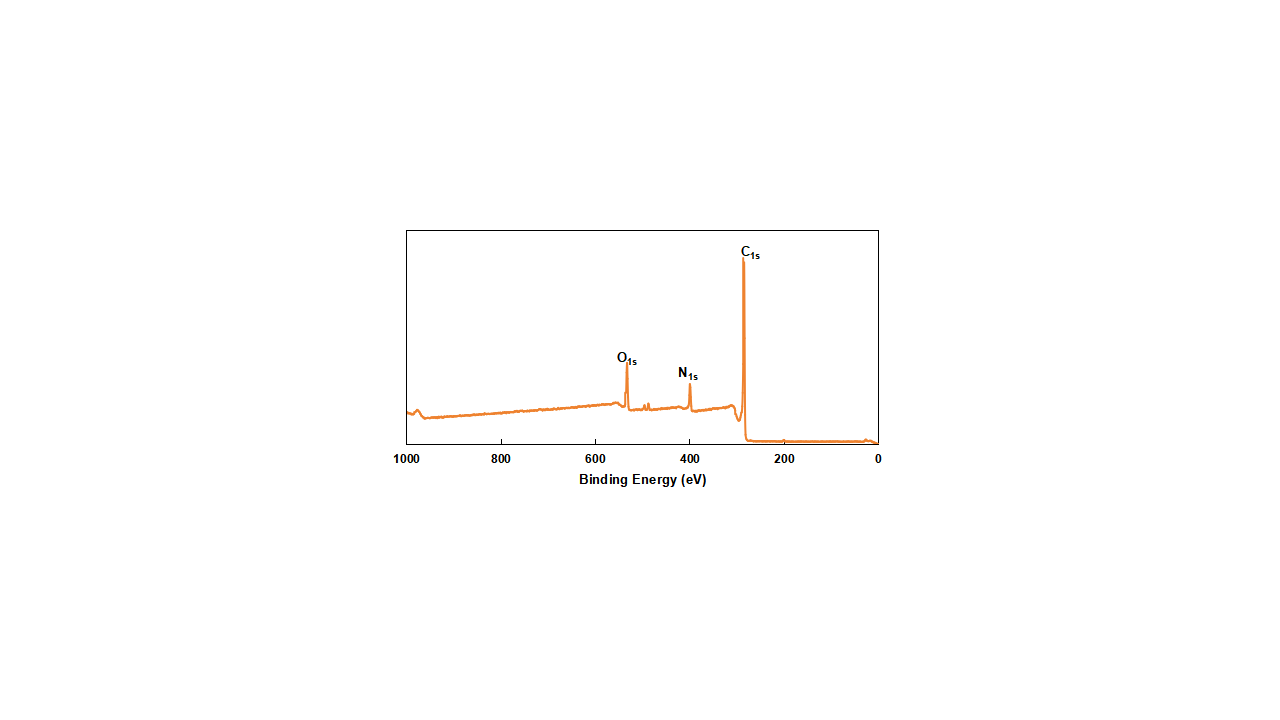


Figure S2 XPS result of synthesized micro-size COF.


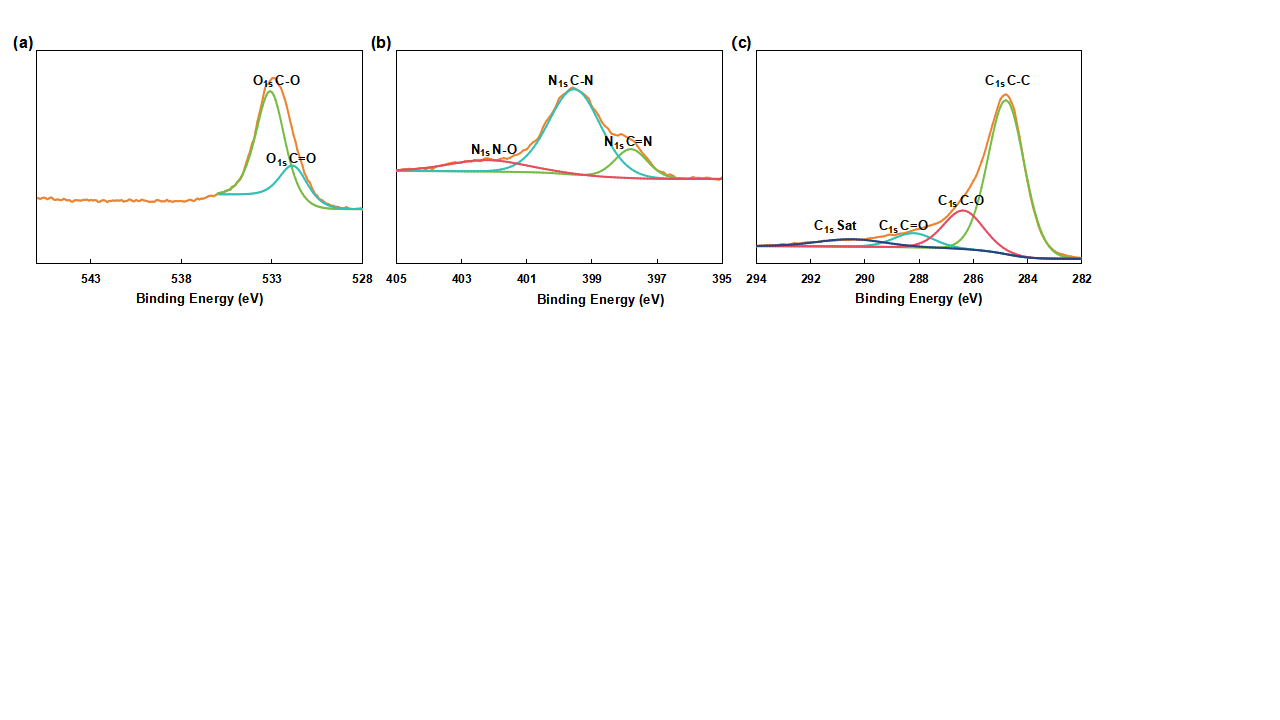


Figure S3 O1s, N1s, and C1s spectrum of mCOF.


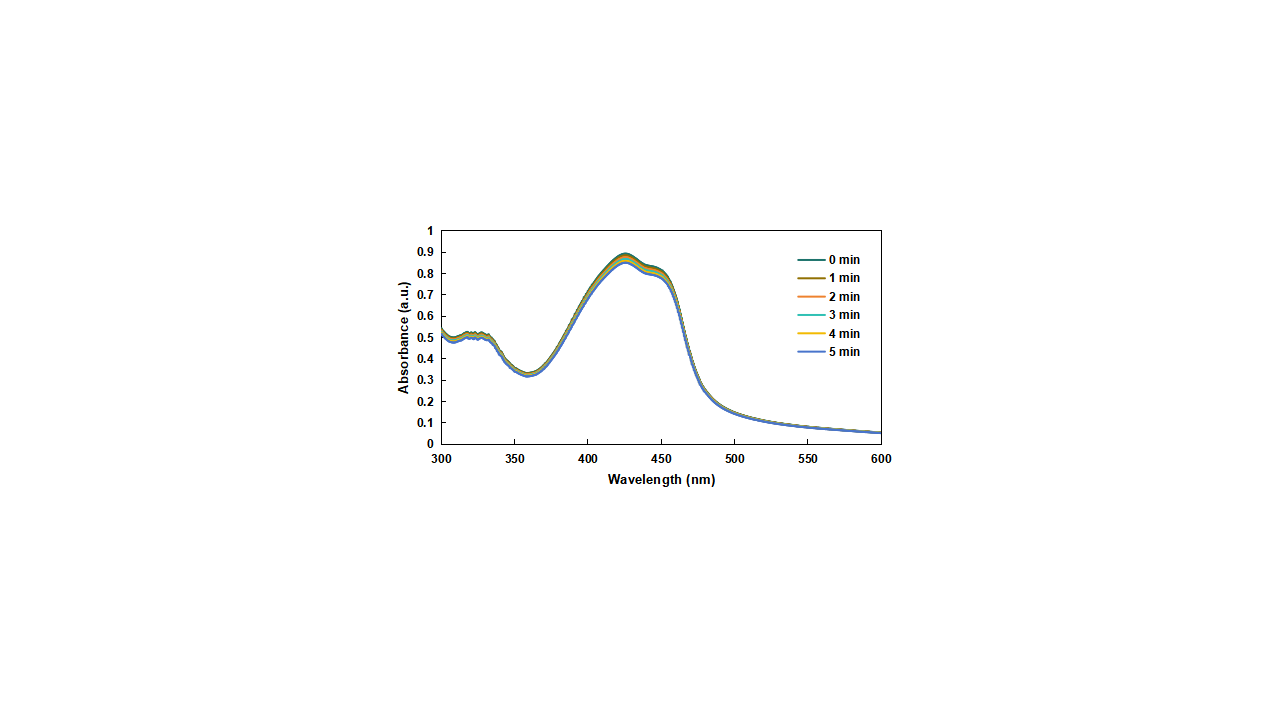


Figure S4 Time-dependent degradation of DPBF by PBS after ultrasonic treatment.


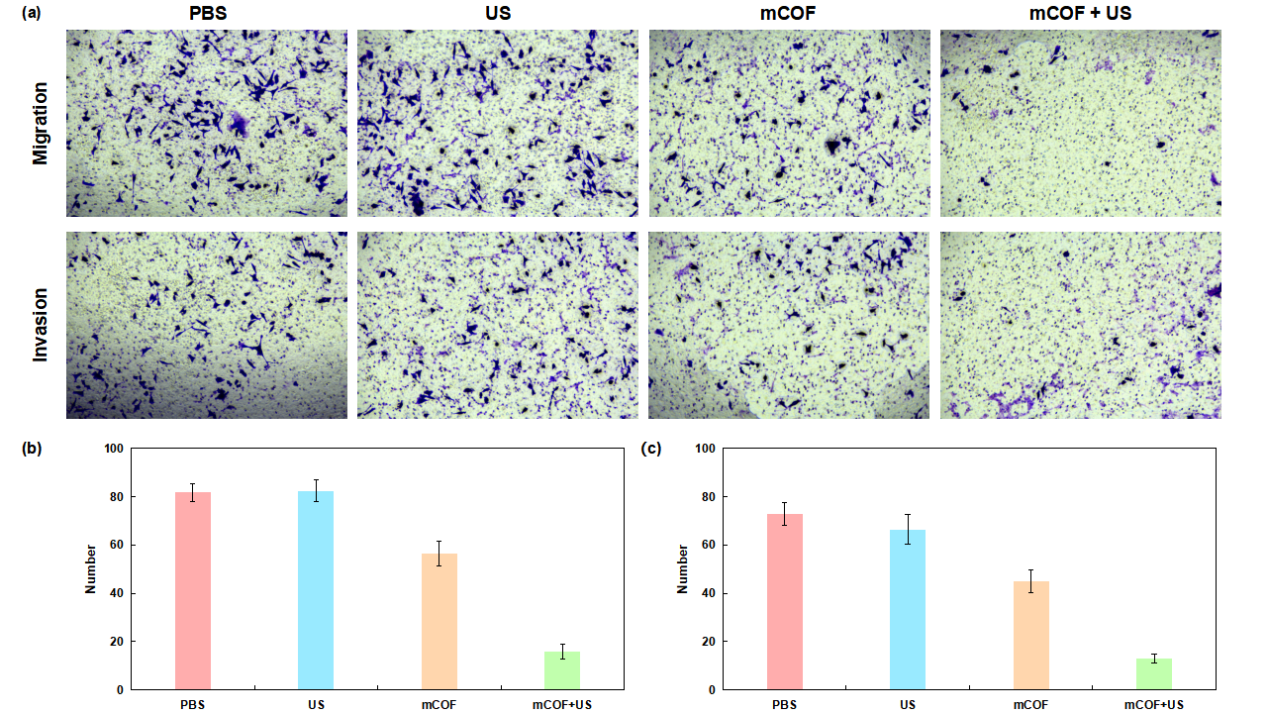


Figure S5 (a) Migration assay and invasion assay results of cells treated with different treatment; (b) the average number of migrated 4T1 cells treated with different treatment; (c) the average number of invasive 4T1 cells treated with different treatment.


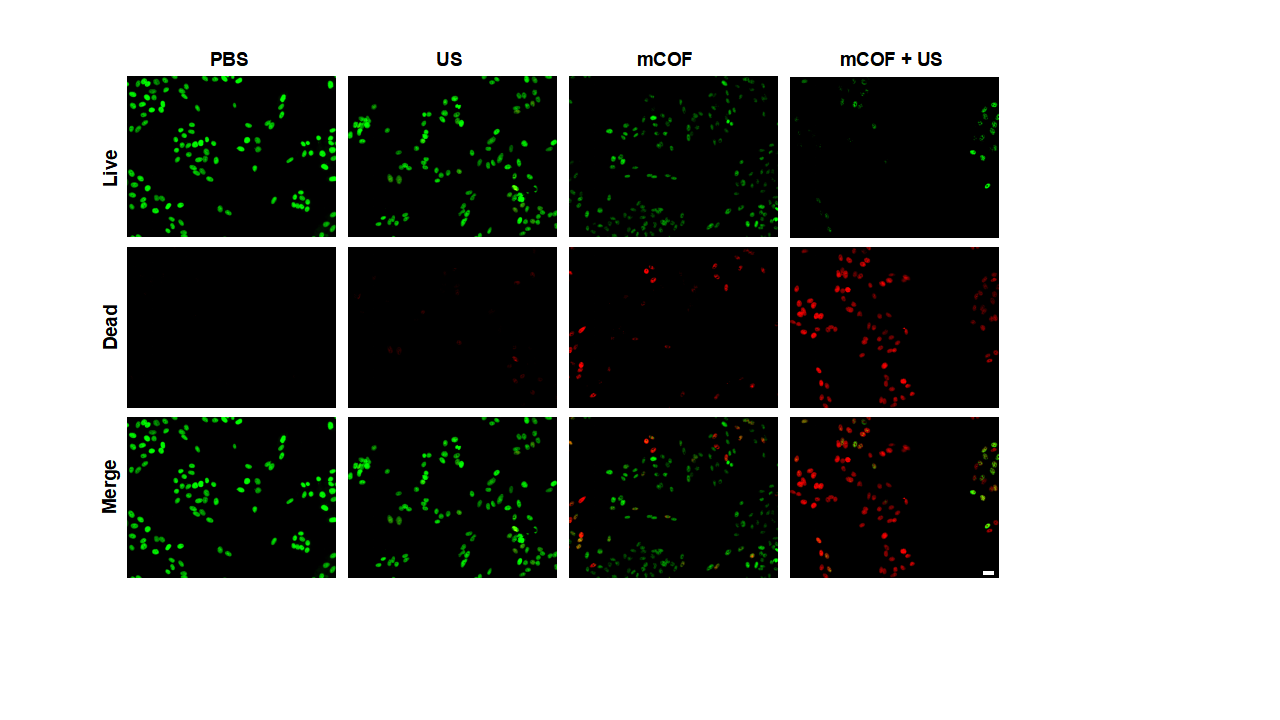


Figure S6 Representative Calcein-AM/PI staining images in 4T1 cells (Scale bar: 100 µm).


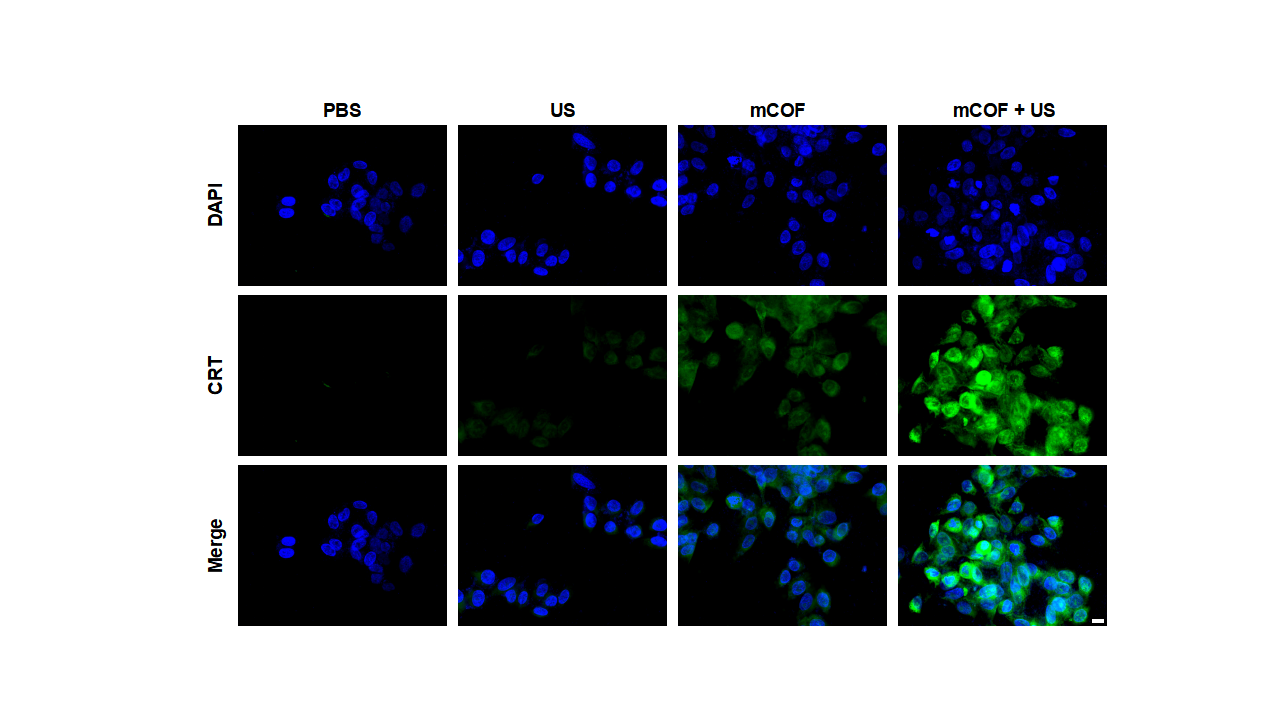


Figure S7 Immunofluorescence staining for CRT in 4T1 cells after various treatments. (Scale bar: 50 µm)


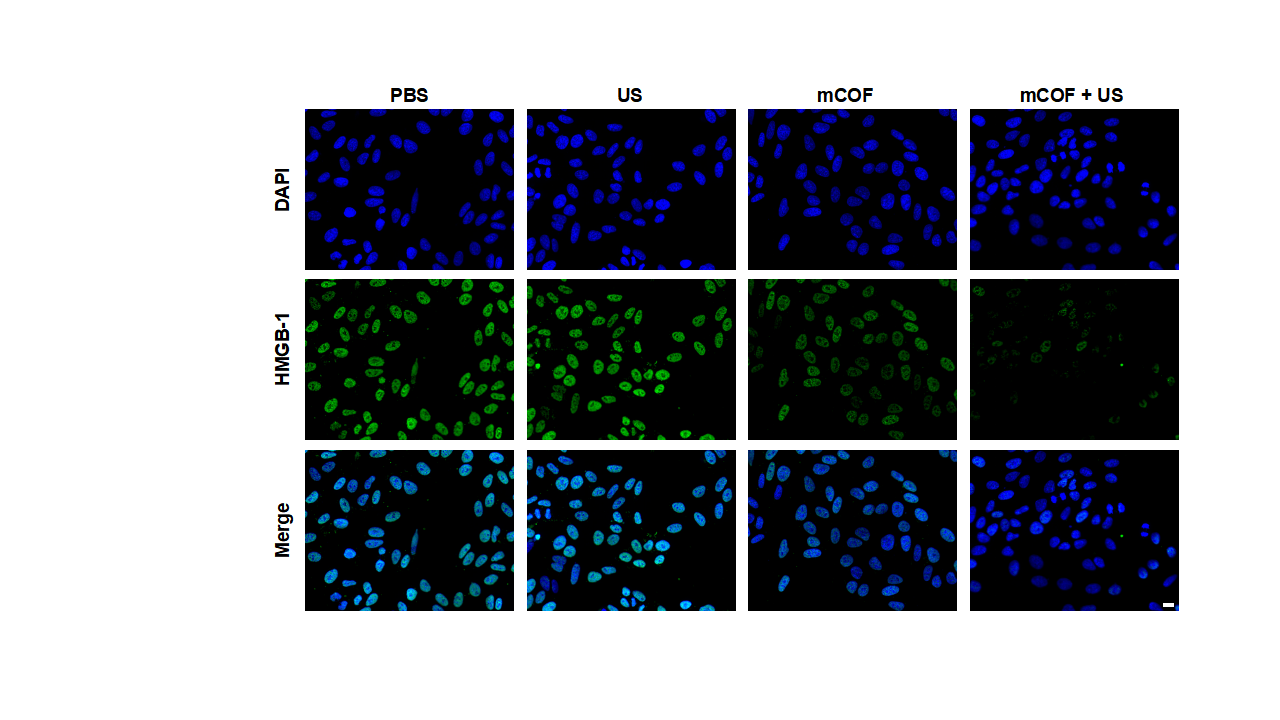


Figure S8 Immunofluorescence staining for HMGB-1 in 4T1 cells after various treatments. (Scale bar: 50 µm)


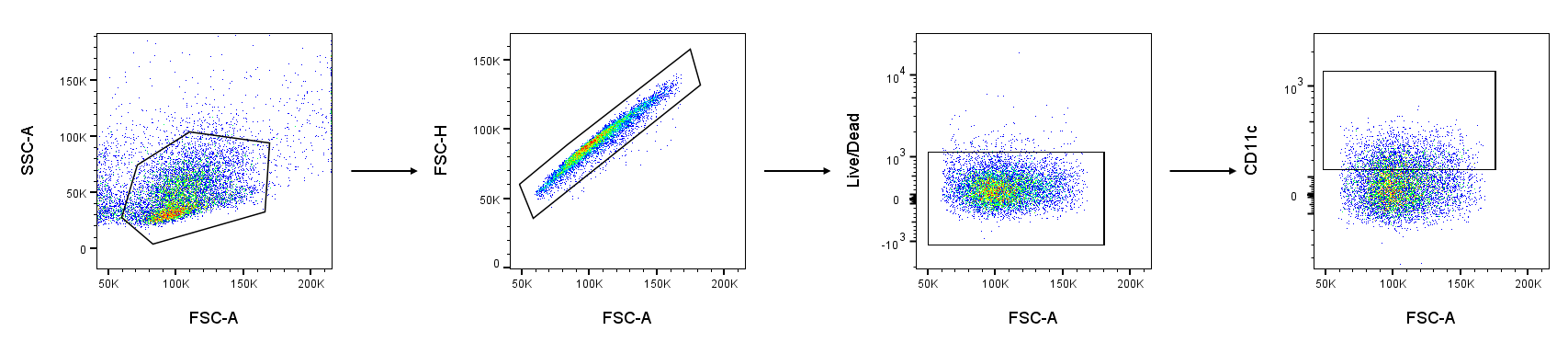


Figure S9 Flow cytometry gating strategy for the analysis of expression of CD80, CD86 costimulators on DCs presented on Figure 6e.


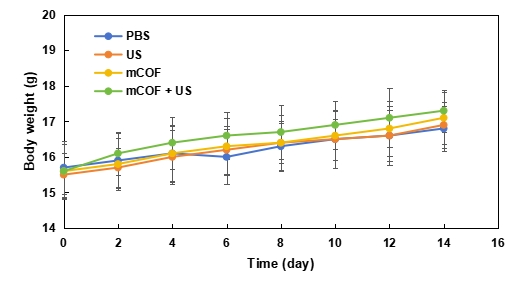


Figure S10 Body weight change of 4T1 tumor-bearing mice receive different treatments. (n=5, mean ± SD)


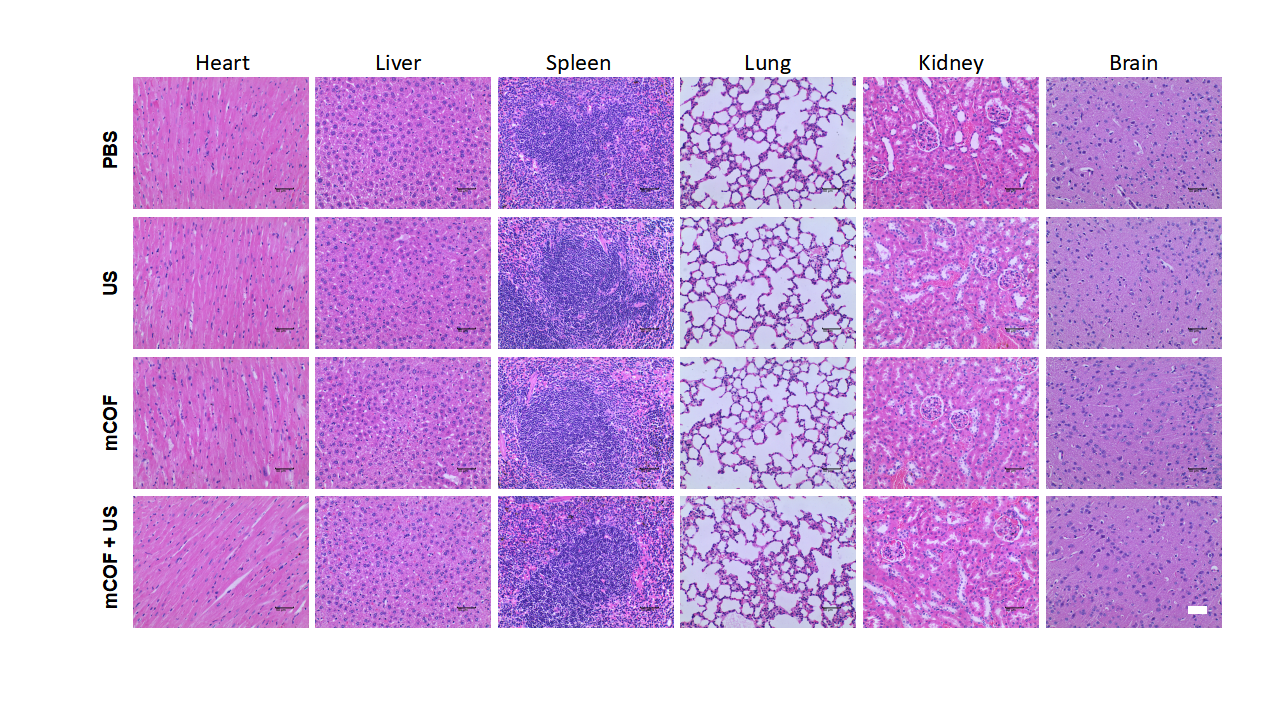


Figure S11 H&E staining of major organs (Heart, Liver, Spleen, Lung, Kidney and Brain) of 4T1 tumor-bearing mice after treatments. (Scale bar: 50 µm)


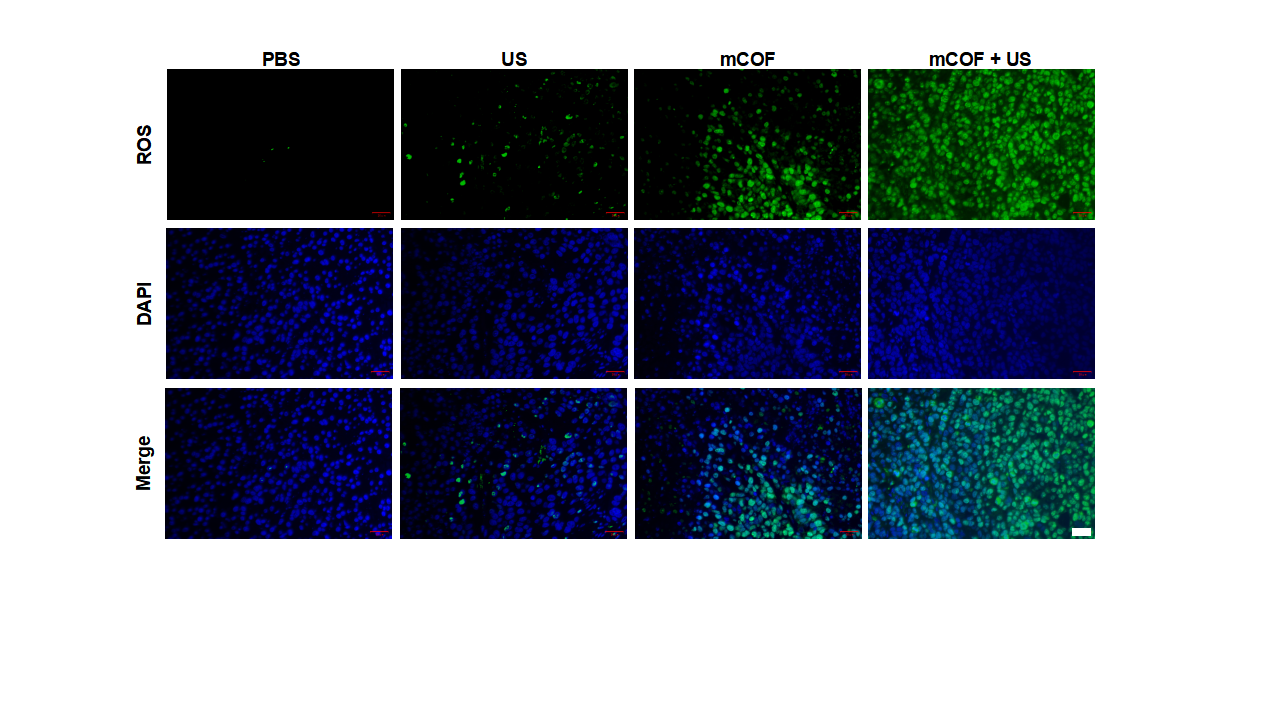


Figure S12 ROS levels in tumors from treated mice. (scale bar: 20 µm)


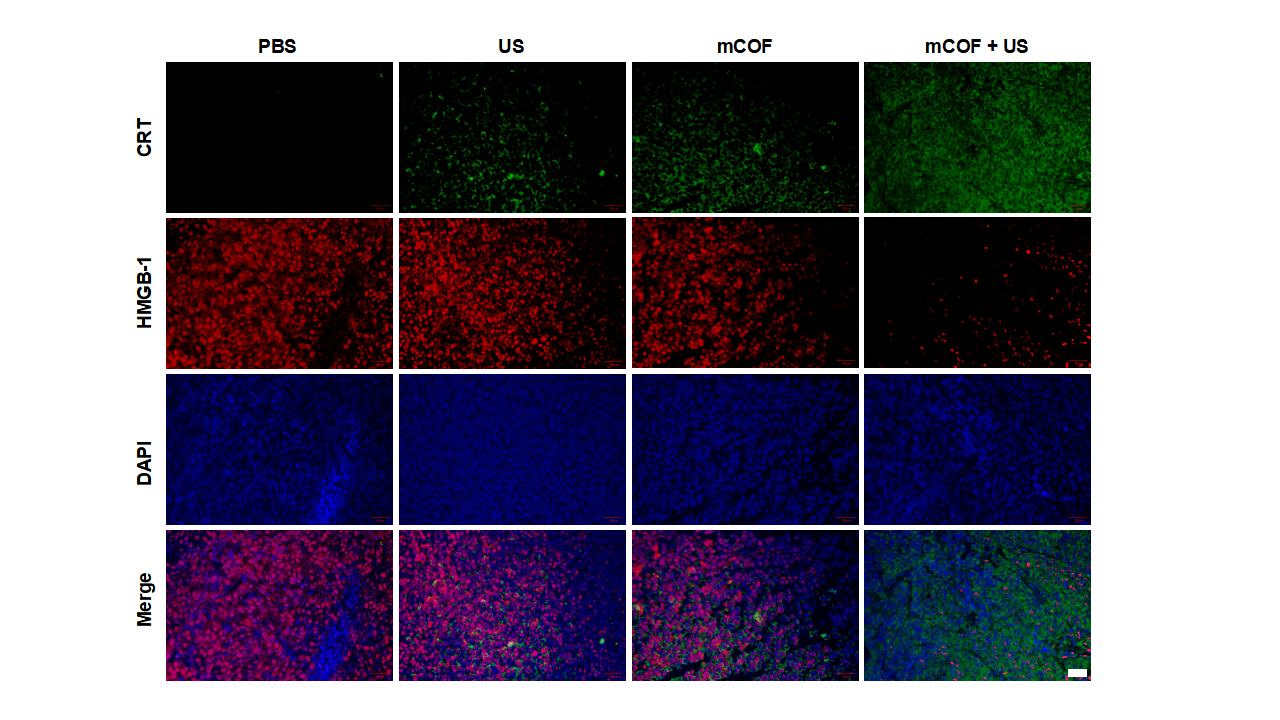


Figure S13 CRT and HMGB1 expression at tumor sites in 4T1 tumor-bearing mice after treatments. (Scale bar: 20 µm)


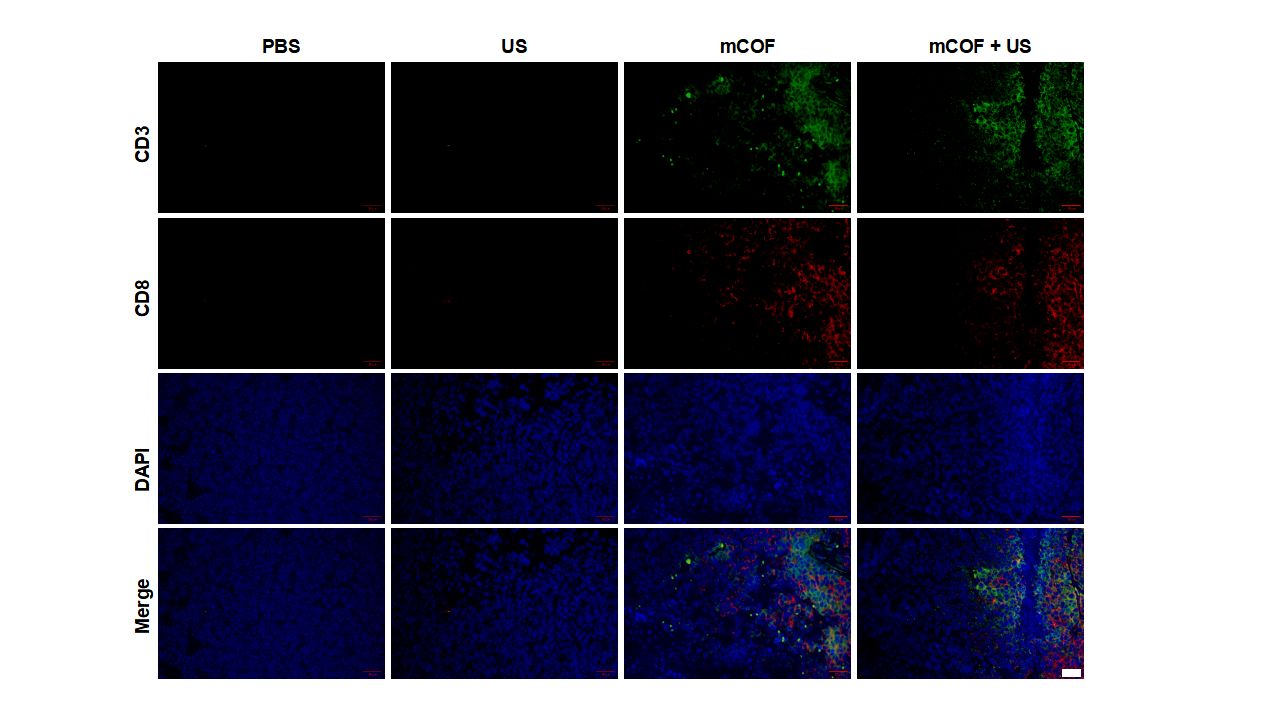


Figure S14 CD3&CD8 expression at tumor sites in 4T1 tumor-bearing mice after treatments. (Scale bar: 20 µm).

Table S1. Quantitative analysis result of figure 5e

| Relative expression of Proteins | Bcl-2 | C-caspase3 | H2AX-S139 | Survivin |
| --- | --- | --- | --- | --- |
| PBS | 0.727 | 0.286 | 0.337 | 0.536 |
| US | 0.787 | 0.326 | 0.345 | 0.658 |
| mCOF | 0.593 | 0.398 | 0.520 | 0.545 |
| mCOF + US | 0.379 | 0.561 | 0.808 | 0.434 |

Table S2. Quantitative analysis result of figure 5f

| Relative expression of Proteins | GPX4 | SLC7A11 | ACSL4 |
| --- | --- | --- | --- |
| PBS | 0.976 | 0.799 | 0.851 |
| US | 1.026 | 0.844 | 0.804 |
| mCOF | 0.612 | 0.465 | 1.228 |
| mCOF + US | 0.335 | 0.237 | 1.506 |
